# Integrated RNA Sequencing Analysis Revealed Early Gene Expression Shifts Associated with Cancer Progression in MCF-7 Breast Cancer Cells Cocultured with Adipose-Derived Stem Cells

**DOI:** 10.3390/cimb46110702

**Published:** 2024-10-23

**Authors:** Minh Ngoc Vu, Hoang Duc Le, Thi Tien Vu, Trung Nam Nguyen, Hoang Ha Chu, Van Ngoc Bui

**Affiliations:** 1 Institute of Biotechnology (IBT), Vietnam Academy of Science and Technology (VAST), Hanoi 100000, Vietnamchuhoangha@ibt.ac.vn (H.H.C.); 2 University of Science, Vietnam National University (VNU-HUS), Hanoi 100000, Vietnam; 3 Faculty of Biotechnology, Graduate University of Science and Technology (GUST), Vietnam Academy of Science and Technology (VAST), Hanoi 100000, Vietnam

**Keywords:** breast cancer, adipose-derived stem cells, RNA sequencing, transcriptomic profiling, weighted gene coexpression network analysis, cancer recurrence

## Abstract

Breast cancer remains a prevalent global health challenge, with tumor-removal surgeries being among the most common treatments but often leading to aesthetic defects. Adipose-derived stem cell (ADSC)-enriched fat grafting in breast reconstruction offers promising therapeutic benefits. However, concerns about its oncological safety persist, particularly regarding the potential risks of promoting cancer recurrence. This study investigated the effects of ADSCs on breast cancer progression by coculturing ADSCs with the MCF-7 breast cancer cell line for a short cell cultivation period of 3 days. We performed an RNA-seq analysis to identify significant transcriptomic changes in cocultured MCF-7 cells and carried out functional enrichment analyses to uncover key biological pathways influenced by ADSCs. Our findings revealed that transcriptomic alterations in MCF-7 cells are linked to aggressive cancer traits, including the upregulation of epithelial–mesenchymal transition (EMT) and the HIF-1 signaling pathway, which indicate a shift toward aerobic glycolysis. Some of the observed gene expression changes also correlated with relapse risk and mortality. These findings underscore the need for further research to explore the implications of these genes and pathways in driving aggressive cancer phenotypes and assess the safety of ADSCs in clinical settings.

## 1. Introduction

Breast cancer accounts for 31% of female cancers worldwide and is one of the most common causes of cancer-related death [1,2]. Patients may undergo breast-conserving surgery, a procedure that removes tumors along with some surrounding healthy tissue, resulting in significant physical and psychological concerns. Autologous fat grafting has emerged as a promising technique to correct tissue defects caused by these surgeries, helping patients regain self-confidence and increasing their quality of life. This procedure involves grafting the patient’s adipose tissue into the breast to correct deformities and asymmetry; however, it suffers from a low retention rate of grafted fat due to complications such as fat resorption and necrosis [3]. In vitro and in vivo experiments have shown that within 24 h of the transplantation of nonvascularized fat tissue, a significant portion of adipocytes, endothelial cells, and hematopoietic cells undergo necrosis and apoptosis and are then replaced by differentiated cells derived from adipose-derived stem cells (ADSCs) and progenitor cells, which have much longer survival rates [4]. To address these challenges, a novel approach called cell-assisted lipotransfer (CAL), which involves enriching adipose tissue with ADSCs before injecting it into the target area, was developed [5]. Its outstanding effects, including elevated graft volume, improved retention rate, and satisfactory aesthetic outcomes, have been reported in many trials [5,6,7].

Despite evidence supporting the use of ADSC-enriched fat grafts, scientific research has not reached a conclusive agreement regarding the oncological safety of grafted ADSCs in breast reconstruction following tumor removal surgeries. After breast-conserving surgery, residual breast cancer cells (BCCs) may remain in the surrounding native tissue [8]. Thus, the primary question is whether ADSCs might stimulate cancer recurrence or even increase the risk of de novo cancer development [9]. These concerns arise from the qualities that make ADSCs attractive for breast reconstruction and tissue augmentation, specifically their differentiation capacity, paracrine release of growth factors and cytokines, and ability to promote vascularization [10].

Research has been conducted to investigate the roles of ADSCs in breast cancer progression and recurrence. ADSCs secrete immunosuppressive cytokines such as IL-6, IL-8, and TGF-*β*1, which inhibit local immune reactions against cancer cells, thus enabling cancer to progress toward more aggressive phenotypes [11]. This finding was supported by the work of Koellensperger et al. (2017), who demonstrated that in a coculture model, ADSCs increased the migration abilities of the low-grade malignant MCF-7 cell line and highly aggressive MDA–MB-231 cells by 11% and 23%, respectively, while increasing tube formation [12]. Wang et al. (2017) confirmed these observations, showing that ADSCs release proangiogenic cytokines and promote tumor growth in a nude xenograft model [13]. Other in vivo experiments in mice also reported an increased metastatic potential of BCCs cotransplanted with ADSCs [14,15].

However, the antitumor effects of ADSCs have also been reported. One study showed that ADSC-conditioned medium (ADSC-CM) induced DNA damage, suppressed proliferation, and triggered apoptosis in MCF-7, MDA-MB-231, and MDA-MB-468 breast cancer cells [16]. Similar outcomes were reported in a different study, in which ADSC-CM decreased the viability of MCF-7 and MDA-MB-231 cells while reducing the migration rate of MCF-7 cells, and the effects persisted even when ADSC-CM proteins were denatured [17]. Sun et al. (2009) reported that ADSCs decreased tumor size and lung metastases by 50% in NOD/SCID mouse models of breast cancer [18]. Clinical investigations have also provided favorable support for the safety of ADSC-based therapies. A clinical trial from 2006 to 2017 demonstrated that no signs of cancer recurrence were detected during a 5-year follow-up period in patients who underwent CAL breast reconstruction [19]. This finding is consistent with a report by Pérez-Cano et al. (2012) in the RESTORE-2 trial that followed 60 patients for one year after CAL procedures [20]. However, several reviews have noted the weaknesses of these trials, including small population sizes, limited control cases, the inclusion of only low-risk patients, and short follow-ups [8,11]. Nevertheless, the consensus among researchers is that the debate remains unresolved. Further comprehensive studies, along with well-designed and long-term trials, are needed to shed light on the safety of ADSC-based therapies in breast cancer treatment [11].

To better understand the benefits and risks of ADSC applications, investigating the molecular interactions between ADSCs and BCCs is crucial. This study aims to provide a comprehensive outlook on the effects of ADSCs on MCF-7 cells at the transcriptomic level via RNA sequencing. A coculture system of MCF-7 cells and ADSCs was established, followed by RNA-seq analysis, differential gene expression, and a weighted gene coexpression network analysis (WGCNA). A protein–protein interaction (PPI) network analysis was also performed to identify gene sets linked to the altered phenotypes of MCF-7 cells in the presence of ADSCs. Additionally, extensive pathway and functional enrichment analyses were conducted to explore the alterations in the biological processes of MCF-7 cells affected by ADSCs. Moreover, disease-free survival analysis via the Gene Expression Omnibus (GEO) database was used to assess the correlation between the identified gene sets and breast cancer relapse, whereas overall survival analysis via The Cancer Genome Atlas (TCGA) data was used to evaluate the link between these gene sets and patient survival outcomes. These results support the current knowledge of the complex roles of ADSCs in regulating breast cancer progression.

## 2. Materials and Methods

### 2.1. Coculturing of MCF-7 Cells and ADSCs

Human breast cancer MCF-7 cells were obtained from ATCC (Manassas, VA, USA), and adipose-derived stem cells (ADSCs) were purchased from Lonza (code PT-5006, Lonza, Switzerland). The cells were cultured in Dulbecco’s modified Eagle’s medium (Sigma–Aldrich, St. Louis, MO, USA) supplemented with 10% fetal bovine serum (FBS), fibroblast growth factor (1 ng/mL), penicillin (100 units/mL), and streptomycin (100 µg/mL) (Corning Inc., Corning, NY, USA). Cultures were maintained in a humidified incubator at 37 °C with 5% CO_2_. At passage 8, the cells were trypsinized, washed with phosphate-buffered saline (PBS), and prepared for coculture. Coculturing was performed using 12 mm transwell culture plates (Sigma–Aldrich, St. Louis, MO, USA) according to our previously described protocol [21]. ADSCs were seeded at a density of 1.5 × 10^5^ cells/mL onto polycarbonate membrane inserts with a pore size of 0.4 µm, while MCF-7 cells were seeded at 5 × 10^4^ cells/mL onto the bottom of the culture plates. MCF-7 cells seeded at the same density in monoculture served as controls. All the experiments were conducted in triplicate, resulting in six samples for RNA sequencing.

### 2.2. RNA Extraction and Sequencing

After three days of culture, total RNA was extracted from cocultured and control (monocultured) MCF-7 cells using a TRIzol reagent kit (Thermo Fisher Scientific, Waltham, MA, USA). The RNA concentration was measured using a BioSpec-nano spectrophotometer (Shimadzu, Kyoto, Japan). Library preparation and paired-end sequencing were carried out by Novogene Co., Ltd. (Beijing, China). Briefly, messenger RNA was isolated using poly-T oligo-attached magnetic beads. The mRNA was then fragmented, and the first and second-strand cDNA were synthesized using random hexamer primers. The construction of cDNA libraries, which involved end repair, A-tailing, and adapter ligation, was performed. Subsequently, polymerase chain reaction (PCR) amplification and purification of the cDNA libraries were conducted. The libraries were pooled and sequenced on the Illumina NovaSeq 6000 platform (San Diego, CA, USA), generating 80–90 million paired-end reads per sample, with a read length of 150 bases.

### 2.3. Sequence Processing

The quality of the sequences was assessed using *FastQC* (v0.12.1) [22] and the results of all the samples were combined using *MultiQC* (v1.21) [23]. *Cutadapt* (v4.8) [24] was then used to filter out sequences that contained adapters and ambiguous bases. Next, the reads were aligned to the GrCh38 reference genome (release 84) using *HISAT2* (v2.2.1) [25]. *Qualimap* (v2.3) [26] was then applied to summarize the coverage profile for each sample. Finally, gene annotation and quantification were performed using *featureCounts* (v2.0.6) [27] with Ensembl annotation. All command-line software was executed on a MacOS 13.4 operating system utilizing 4 computational threads for parallel processing and managed via Anaconda version 23.7.3.

### 2.4. Differential Gene Expression Analysis

The differential gene expression analysis was performed in R programming language version 4.3.1. First, the gene counts were processed according to Bioconductor’s *edgeR* pipeline (v3.42.4) [28]. All genes with a count-per-million (CPM) value lower than 10 in more than three samples were discarded. The count data were modeled using a negative binomial distribution. Then, *p* values from the exact test for differential expression in cocultured versus mono-cultured MCF-7 cells were adjusted for the false discovery rate (FDR) according to the Benjamin–Hochberg method [29]. Differentially expressed genes (DEGs) were identified using thresholds of FDR < 0.05 and |log_2_FC| > 1.

### 2.5. Weighted Gene Coexpression Network Analysis (WGCNA)

Weighted gene coexpression network analysis (WGCNA) was carried out to identify coexpressed gene modules associated with the phenotypes of cocultured cancer cells. For this analysis, the R package *WGCNA* (v1.72–5) was used [30]. A soft-thresholding power (*β*) was selected based on mean connectivity and scale-free topology fit, which were calculated by the *pickSoftThreshold* function. Average-linkage hierarchical clustering using a topological overlap matrix-based dissimilarity measure was applied to all genes to classify them into distinct modules. Highly similar modules were merged using the default *mergeCutHeight* threshold of 0.25. Pearson’s correlation coefficient was calculated to assess the relationships between the modules and coculture phenotypes. Gene significance (GS) and module membership (MM), which represent the correlation between module eigengenes and individual gene expression profiles, were calculated for each gene within a module. Genes with GS > 0.9 and MM > 0.9 in the selected module were considered significant for further analysis. Additionally, the intersection between genes in the selected module and DEGs was screened using the *VennDiagram* package (v1.7.3) [31] and included in subsequent analyses.

### 2.6. Functional Enrichment Analysis

Enriched gene functions associated with cocultured MCF-7 cells were identified using the *clusterProfiler* package (v4.8.3) [32]. The functional databases employed in this analysis included Gene Ontology (GO) terms (with all three subcategories, i.e., biological processes, molecular functions, and cellular components) [33], Kyoto Encyclopedia of Genes and Genomes (KEGG) pathways [34], hallmark gene sets from the Molecular Signatures Database (MSigDB) [35], and Reactome pathways [36]. Gene set enrichment analysis (GSEA) was conducted for the gene expression results and module genes with 10,000 permutations. Pathways and functions with FDR < 0.05 were considered significant. Additional filtering was based on “tag” and “signal” values, representing the number of genes that contributed to the enrichment score and strength. Only pathways and functions with tag > 30 and signal > 20 were selected for visualization. For the intersection gene set, which contained a limited number of genes, overrepresentation analysis (ORA) was carried out instead of GSEA. Visualizations were generated using R packages such as *pathview* (v1.40) [37], *enrichplot* (v1.20.3) [38], and *ggplot2* (v3.5.1) [39].

### 2.7. Protein–Protein Interaction Network Analysis

Protein–protein interaction (PPI) networks were constructed for the intersection of the identified gene module and DEGs using the STRING database (v12) [40], with a high confidence score threshold of 0.7. The PPI network was then analyzed in Cytoscape 3.10 with the *CytoBubba* plugin (v0.1) [41]. The Maximal Clique Centrality (MCC) algorithm was used to calculate node centrality, and the 40 nodes with the highest scores were considered intramodular hub genes.

### 2.8. Disease-Free Survival Analysis

To evaluate whether the hub genes are associated with breast cancer relapse, the public dataset GSE2034 from the GEO database was used. This dataset comprises 286 lymph node-negative breast cancer samples, with expression levels of 22,283 transcripts generated by the Affymetrix Human Genome U133A Array [42]. Hierarchal clustering and principal component analysis (PCA) were employed to detect outliers, resulting in three samples being excluded. Among the remaining samples, 106 experienced relapses, whereas 177 were relapse-free (Table S1). The Wilcoxon rank-sum test was applied to compare the expression of the selected gene set between the relapse and relapse-free groups in the ER-positive and ER-negative samples, with a significance threshold of *p* < 0.05. Univariate Cox regression analysis was subsequently used to evaluate the relationship between gene expression and recurrence risk using the R *survival* package (v3.7) [43]. In addition, Kaplan–Meier survival curves were generated via the GEPIA tool on a larger TCGA cohort, with samples classified into low- and high-expression groups based on the median transcript-per-million (TPM) value of each gene [44].

### 2.9. Overall Survival Analysis

The prognostic value of the identified hub genes was evaluated using the data from TCGA-BRCA (https://www.cancer.gov/tcga, accessed on 16 August 2024). The transcriptomic data and clinical characteristics of 927 female breast cancer patients were obtained from the Genomic Data Commons (GDC) portal. After outlier removal, 915 samples were retained for analysis (Table S2). The Wilcoxon rank-sum test was applied to compare the expression of the hub genes across different cancer stages. Cox regression and Kaplan–Meier survival curves were also generated to explore the associations between gene expression and overall patient survival.

## 3. Results

### 3.1. RNA-Seq Data Processing

Approximately 526.8 million reads were produced from six samples. After removing reads with adapters and ambiguous bases, 415.1 million sequences remained, representing 93–93.7% of the reads that met the filtering criteria (Table 1). The MultiQC quality control report indicates a high base call accuracy of over 99.9% across all sequences (Phred scores > 30), along with a stable GC content, duplication rate, and relatively consistent coverage profile across gene positions in all samples (Figure S1). The reads were successfully mapped to the reference genome, with high mapping rates ranging from 96.49 to 97.01% (Table 1). Over 70% of the mapped reads in all samples were assigned to gene IDs. A total of 19,340 genes with at least 10 CPM in more than three samples were retained for downstream analyses.

### 3.2. Gene Expression Profile of Cocultured MCF-7 Cells Compared with That of Monocultured Cells

**Figure 1:**
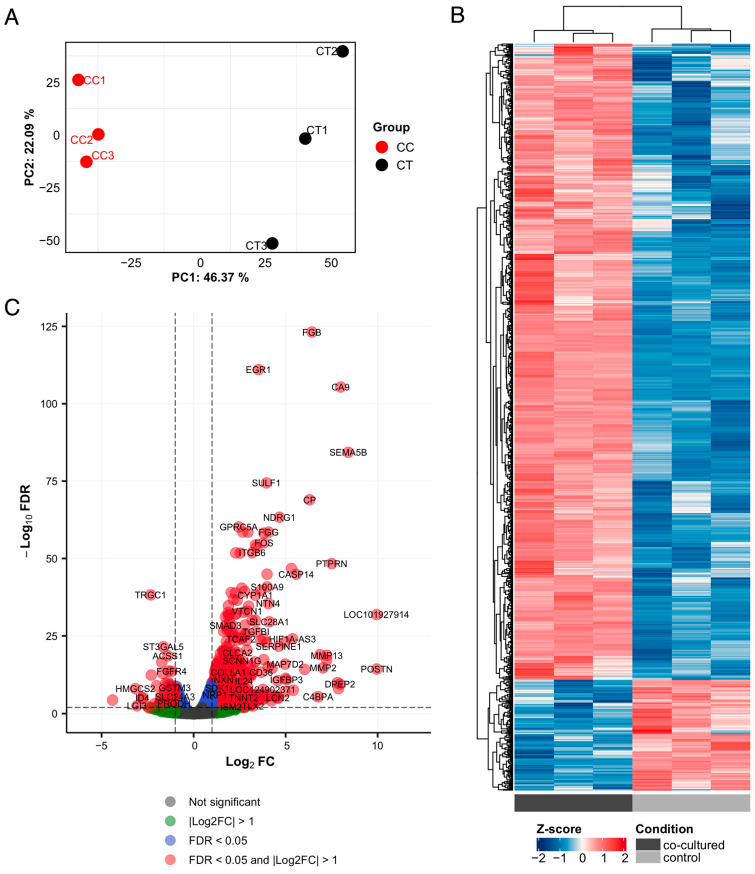
Overview of differential gene expression between cocultured MCF-7 cells and control MCF-7 cells. (**A**) Principal component analysis (PCA) results; CC: coculture; CT: control. (**B**) Hierarchical clustering of DEGs across samples. (**C**) Volcano plot illustrating DEGs with cutoff values set at FDR < 0.05 and |log_2_FC| > 1.

The PCA plot generated based on the gene expression patterns of the samples shows a clear separation between cocultured and monocultured MCF-7 cells (Figure 1A). An overview of gene expression revealed that compared with monocultured cells, MCF-7 cells cocultured with ADSCs presented a distinct expression profile (Figure 1B). Using the thresholds |log_2_FC| > 1 and FDR < 0.05, 952 DEGs were identified; among them, 141 were downregulated, and 811 were upregulated (Figure 1C).

### 3.3. Hub Gene Identification and Coexpression Network Analysis

**Figure 2:**
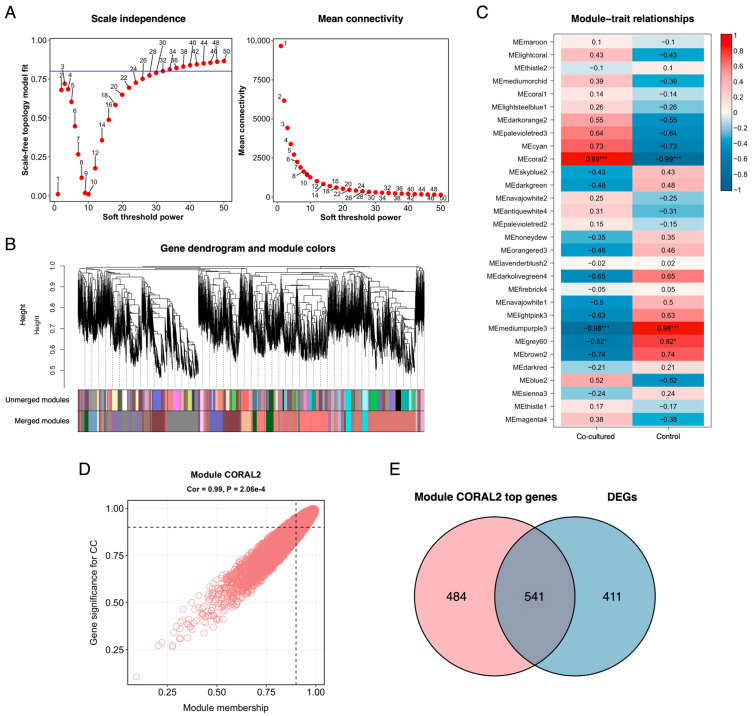
Weighted gene coexpression network analysis results. (**A**) Scale independence and mean connectivity across different soft threshold powers. (**B**) Dendrograms of gene clusters based on topological overlap dissimilarity and module colors. (**C**) Heatmap illustrating the correlation of gene modules with cocultured and controlled MCF-7 cells. ***: *p* < 0.001; *: *p* < 0.05. (**D**) Gene significance and module membership values of genes in the *Coral2* module. (**E**) Number of unique and common genes between the *Coral2* module and the DEG list.

WGCNA was performed to identify modules of coexpressed genes associated with cocultured MCF-7 samples. A *β* value of 30 was chosen based on scale independence and mean connectivity plots, resulting in 30 distinct modules (Figure 2A,C). The *Coral2* module, comprising 3596 genes, presented the highest Pearson correlation with the cocultured samples (*r* = 0.99, *p* < 0.001). Figure 2D shows a strong positive correlation between gene significance (GS) and module membership (MM) for all genes within this module. A total of 1052 genes with MM > 0.9 and GS > 0.9 (top 30%) were selected for further analysis. Among them, 541 genes were also differentially expressed between the two sample types, as shown in Figure 2E, and this intersection gene set was used for the following step.

### 3.4. The PPI Network of the Top-Ranked Genes Revealed Distinct Subnetworks

**Figure 3:**
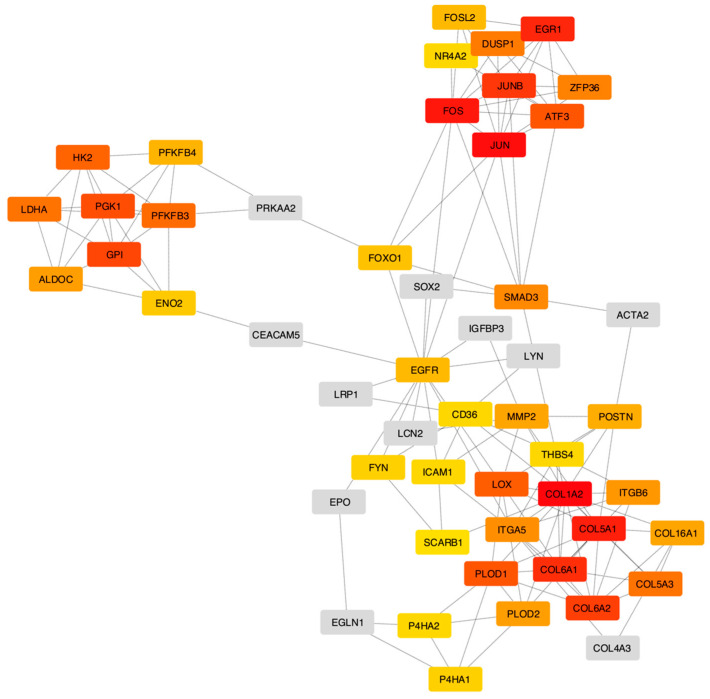
Network visualization of the 40 top-ranked hub genes and their expanded interactions identified by the MCC algorithm. Hub genes are color-coded, with red indicating the highest-ranked genes. The expanded nodes are shown in gray.

The selected gene set was utilized to construct a rank-based (PPI) network. Figure 3 shows that the hub genes formed a highly interconnected network, with COL1A2, JUN, FOS, COL5A1, and EGR1 serving as central nodes of distinct subnetworks. The largest subnetwork centers around collagen family members and includes cell–cell adhesion molecules such as ICAM1, FYN, ITGA5, and ITGB6. Another subnetwork comprises glycolysis- and hypoxia-related genes, including PGK1, HK2, and LDHA. FOS, JUNB, JUN, and EGR1 are involved in various biological processes, including the cell cycle and differentiation [45].

### 3.5. Functional Enrichment Analysis Revealed Activated Pathways Associated with Cancer Progression

**Figure 4:**
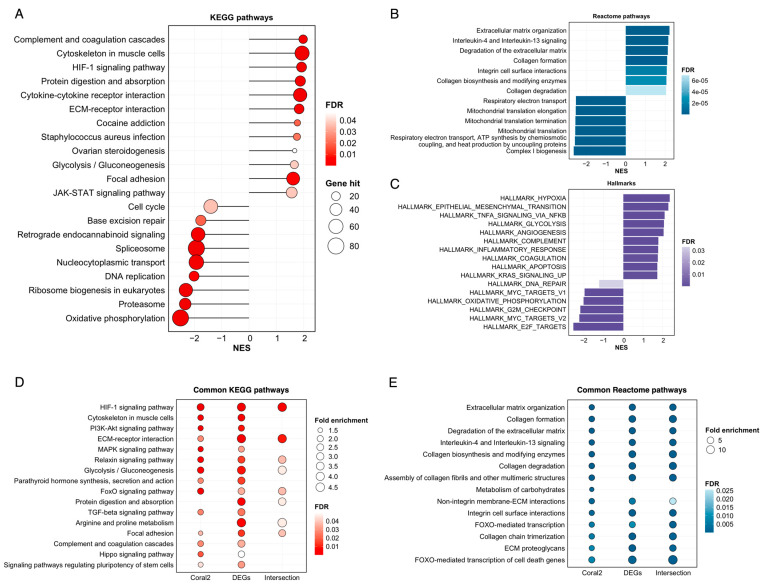
Functional enrichment analysis results. (**A**) Most significantly enriched KEGG pathways, (**B**) Reactome pathways, and (**C**) hallmarks identified via GSEA on the overall gene expression profile. (**D**) Overrepresented KEGG pathways and (**E**) Reactome pathways detected in the *Coral2* gene module, differentially expressed genes (DEGs), and their intersection (NES: normalized enrichment score, FDR: false discovery rate).

We employed a functional enrichment analysis of various gene sets to comprehensively assess the altered functions and pathways that may influence MCF-7 cell behaviors. GSEA was first applied to all genes regardless of their significance levels to provide an overview of functional alterations in cocultured MCF-7 cells. Figure 4A shows that the most significantly enriched KEGG pathways included those related to immune responses, such as “complement and coagulation cascade” and “cytokine–cytokine receptor interaction” (NES > 1.8, FDR < 0.001). Similarly, “Interleukin-4 and interleukin-3 signaling” was also the top enriched Reactome pathway (NES > 2, FDR < 0.001), which is consistent with “complement” and “inflammatory response” in the hallmark analysis results (Figure 4B,C). Other pathways, such as “protein digestion and absorption”, “ECM–receptor interaction”, “focal adhesion”, and “extracellular matrix organization” or “integrin cell surface interaction”, suggested substantial changes in the interactions of MCF-7 cells with extracellular matrix components (Figure 4A,B). Several downregulated pathways are related to the principal processes of the cell cycle, i.e., “base excision repair”, “DNA replication”, “mitochondrial translation”, etc., which are confirmed by the enriched cell cycle hallmarks “DNA repair” and the “G2M checkpoint” (Figure 4A,C). Hallmark analysis also revealed the upregulation of epithelial–mesenchymal transition (EMT), which is a signature process in invasive breast cancer. The overrepresented pathways were also consistently detected across different gene sets (Figure 4D,E).

**Figure 5:**
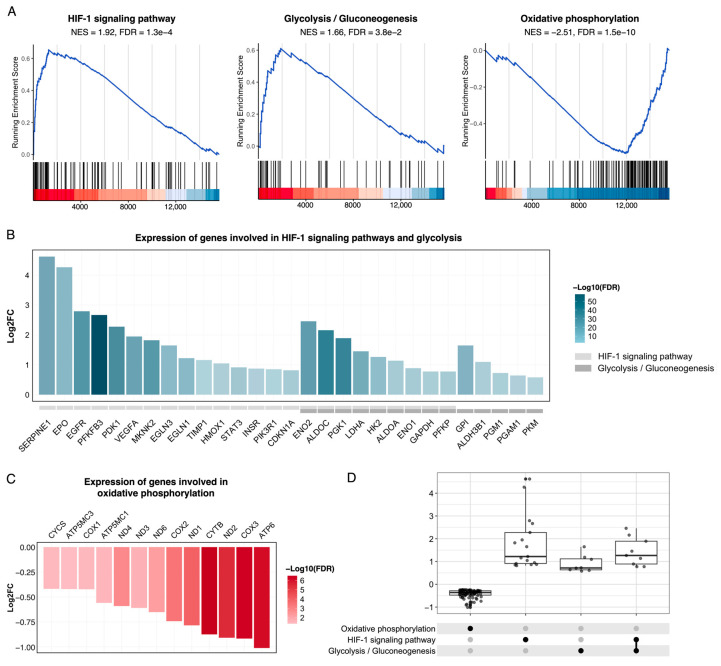
Enrichment of the HIF-1 signaling pathway, glycolysis/gluconeogenesis, and oxidative phosphorylation in cocultured MCF-7 cells. **(A**) Running enrichment score. (**B**) Differential expression of genes associated with the HIF-1 signaling pathway and glycolysis. (**C**) Differential expression of genes linked to oxidative phosphorylation. (**D**) UpSet plot showing the distribution of fold changes in genes uniquely present in each selected pathway and those shared across pathways.

In addition, the upregulation of the HIF-1 signaling pathway and glycolysis/gluconeogenesis KEGG pathways, as well as the hypoxia and glycolysis hallmarks, were interesting results, as MCF-7 cells were cocultured with ADSCs at a sufficient oxygen level (Figure 5A). The HIF-1 signaling pathway directly orchestrates the response of cells to hypoxia, which is an enriched GO biological process term (Figure S2). On the other hand, the downregulation of the “oxidative phosphorylation”, “proton transmembrane transport”, “mitochondrial transmembrane transport”, and “proton motive force-driven ATP synthesis” terms (NES < -2.68) suggested a reduction in ATP synthesis via the electron transport chain in cocultured MCF-7 cells (Figure S2). A more thorough screening of the expression of genes involved in these processes revealed significant upregulation of key hypoxia response genes (Figure 5B). These genes include VEGFA (2^1.9^-fold), EPO1 (2^4.3^-fold), and SERPINE1 (2^4.6^-fold), which play crucial roles in angiogenesis, and LDHA (2^1.5^-fold) and GAPDH (2^0.8^-fold), which are responsible for ATP synthesis via glycolysis. The specific roles and regulators of these genes in the HIF-1 signaling pathways are presented in Figure S3. Moreover, genes involved in oxidative phosphorylation (e.g., ATP6, COX3, and CYTB) were mildly downregulated (Figure 5C). These findings suggest a metabolic shift toward aerobic glycolysis in cocultured cells, an established hallmark of cancer malignancy.

### 3.6. Disease-Free Survival Analysis and the Wilcoxon Test Revealed Genes Differentially Expressed in Relapsed Tumor Samples

**Figure 6:**
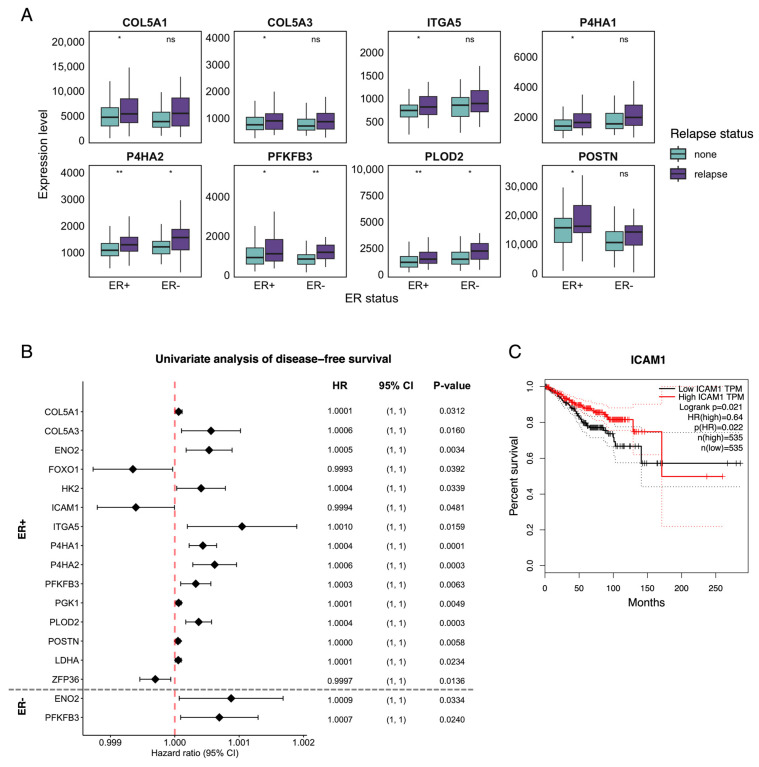
Disease-free survival analysis results. (**A**) Differential expression of genes between relapse-free and relapsed tumor samples in ER+ and ER- breast cancer subtypes within the GSE2034 dataset (**: *p* < 0.01, *: *p* < 0.05, ns: not significant). (**B**) Univariate Cox regression analysis of gene expression for predicting relapse status in ER+ and ER- breast cancer patients from the GSE2034 dataset (CI: confidence intervals). (**C**) Kaplan–Meier analysis of the impact of ICAM1 expression on disease-free survival in breast cancer patients from the TCGA cohort analyzed via GEPIA.

We then assessed whether the expression of the identified significant genes is associated with cancer relapse using the GSE2034 dataset, which includes RNA-seq data of relapse-free and relapsed tumor tissues from lymph node-negative breast cancer patients. The nonparametric Wilcoxon test revealed that 8 out of 40 hub genes were differentially expressed between the relapsed and nonrelapsed samples (Figure 6A). In particular, the expression of P4HA2, PFKB3, and PLOD2 was elevated in relapsed ER+ and ER- tumors, whereas the expression of COL5A1, COL5A2, ITGA5, P4HA1, and POSTN only exhibited significant differences in relapsed ER+ tumors.

To further assess the prognostic relevance of these hub genes, univariate Cox regression analysis was conducted to calculate hazard ratios (HRs) for relapse risk. For ER+ tumors, most hub genes had HR values between 0.9993 and 1.001, suggesting a minimal influence on relapse probability and no strong prognostic impact (Figure 6B). Similar trends were observed for the ER- samples; however, the confidence levels were much lower because of the limited number of data points. We did not perform multivariate analysis on this dataset due to insufficient clinical data. Nevertheless, we validated our findings using GEPIA, which uses a larger TCGA cohort of 1070 patients, and performed Kaplan–Meier analysis along with HR calculations. The results revealed that only ICAM1, which was upregulated 2^1.2^-fold in cocultured MCF-7 cells, was significantly associated with relapse probability (HR = 0.64, *p* = 0.02) (Figure 6C).

In addition, we screened the expression changes of genes included in widely recognized prognostic panels, namely, the Oncotype DX Breast Recurrence Score test [46] and the MammaPrint assay [47]. The Oncotype DX Breast Recurrence Score test evaluates the expression of 21 genes by reverse transcription polymerase chain reaction (RT–PCR) in tumor tissues to calculate a recurrence score that guides treatment in early-stage breast cancer [46]. On the other hand, the MammaPrint assay is a microarray-based gene expression profiling tool that analyzes 70 genes to categorize ER-positive and ER-negative tumors into low-risk and high-risk groups of distant metastases [47,48]. In cocultured MCF-7 cells compared with control cells, significant changes in the expression of 6 genes from the Oncotype DX panel were detected, and 13 genes from the MammaPrint assay were significantly differentially expressed (FDR < 0.05) (Figure S4). These findings suggest that ADSCs may influence the prognostic profiles of MCF-7 cells and impact risk classification in clinical settings.

### 3.7. Overall Survival Analysis Showed Hub Genes’ Associations with Mortality

**Figure 7:**
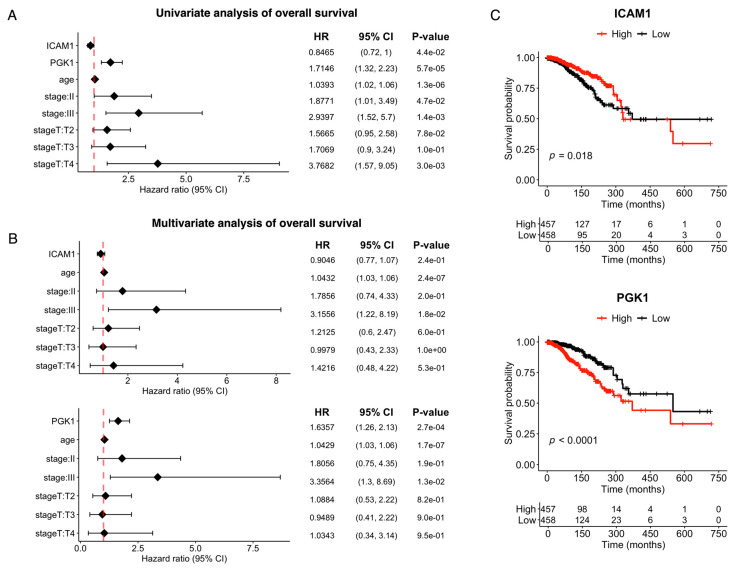
Overall survival analysis results. (**A**) Univariate and (**B**) multivariate Cox regression analyses of gene expression and clinical features predicting the overall survival of breast cancer patients from the TCGA-BRCA dataset. (**C**) Kaplan–Meier analysis of the impact of ICAM1 and PGK1 expression on patient survival.

Wilcoxon rank-sum test was applied to the TCGA-BRCA dataset, and the results revealed that most hub genes in the PPI network were significantly differentially expressed between early pathological stages of breast cancer, particularly between stages I and II and between stages T1 and T2 (Table S3). These findings suggest that these genes may play a role in the progression of early-stage breast cancer. Furthermore, overall survival analysis based on univariate Cox regression was conducted to determine whether the identified hub genes were associated with patient survival probabilities. ICAM1 and PGK1 were the only two genes significantly associated with overall survival (*p* < 0.05). ICAM1 had a hazard ratio (HR) of 0.84, whereas PGK1 had an HR of 1.7, indicating an increased risk of mortality with increased expression (Figure 7A). Additionally, when a multivariate Cox regression model incorporating clinical variables such as age, overall stage, and pathological stage T was used, only PGK1 remained significantly associated with the overall survival (Figure 7B). Kaplan–Meier survival curves confirmed the significance of PGK1, revealing a clear association between high expression levels of the gene and decreased survival probability (Figure 7C). However, for ICAM1, this analysis contradicted the multivariate Cox regression findings and only aligned with the univariate regression results (Figure 7B).

## 4. Discussion

This work focused on the transcriptomic changes in ADSC-cocultured MCF-7 cells, a luminal A subtype that accounts for 40% of all breast cancer cases [49]. Even though MCF-7 cells are poorly aggressive, previous studies have shown that exposure to ADSCs enhances their migration and metastasis properties [12,13]. Nonetheless, limited research has been conducted on the molecular pathways underlying these changes. Utilizing RNA-seq data analysis, this study demonstrated that, compared with monocultured cells, MCF-7 cells cocultured with ADSCs presented a unique gene expression profile, with the expression of most DEGs being upregulated. WGCNA and hub gene identification revealed genes that were significantly correlated with the coculture phenotypes. Network visualization of the 40 top-ranked genes revealed interacting subnetworks involved in glycolysis and angiogenesis, cell adhesion, movement, and interaction with the ECM. These findings are consistent with the functional enrichment results (Figure 4A), which demonstrated enhanced activity of ECM–receptor interactions, protein digestion, the relaxin signaling pathway, the cytoskeleton in muscle cells, and focal adhesion. Changes in the composition of extracellular matrix (ECM) components, such as COL5A1, COL6A1, and COL6A2, along with ECM-bound factors, such as ITGB6, ITGA5, and ITGB5, suggest that ECM remodeling associated with EMT occurs in cocultured MCF-7 cells. This remodeling is crucial for tumor migration and metastasis, as it removes barriers and facilitates the creation of pathways for cellular movement through the surrounding matrix [50]. This finding aligns with the current understanding that ADSCs facilitate EMT in breast cancer, thus driving cells toward more invasive traits [12].

Moreover, the enrichment of hypoxia-response mechanisms according to the GSEA and ORA results, coupled with hypoxia and glycolysis genes in the PPI network, is notable since the MCF-7 cells were cocultured with ADSCs at a sufficient oxygen level. The activation of the HIF-1 signaling pathway, which regulates cellular responses to hypoxia, implied a shift in the energetics of MCF-7 cells when exposed to ADSCs. Normally, HIF-1α is degraded under normoxic conditions by the E3 ubiquitin ligase complex [51]. Since this pathway is activated when the cells are cultured at standard oxygen levels, we hypothesize that the paracrine secretory effects of ADSCs cause HIF-1α in MCF-7 cells to avoid degradation and stabilize. This might be due to the 2^2.4^-fold increase in the expression of P4HA1, which allows HIF-1α to dimerize with HIF-1β in the nucleus to form a HIF-1 heterodimer complex and thereby promote the expression of multiple genes involved in angiogenesis and erythropoiesis to increase oxygen delivery [52]. Our analyses revealed that examples of these genes include VEGFA and EPO (Figure 5B). HIF-1 signaling also drives anaerobic metabolism, with the overexpression of PDK1 (2^2.3^-fold) inhibiting the TCA cycle through phosphorylating PDH, forcing the cells to favor glycolysis for ATP synthesis (Figure S3). Furthermore, the upregulation of key glycolytic genes, including SLC2A3 (or GLUT3, a glucose transporter) and LDHA, was detected (Figure 5B). In glycolysis, LDHA converts pyruvate to lactate, not only allowing energy production but also increasing VEGF expression independently of oxygen levels [53,54]. Notably, EGLN1 (PHD2), a prognostic gene that is significantly overexpressed in cocultured cells (2^1.2^-fold) (Figure S4B), directly induces HIF-1α degradation [51]. We speculate that the elevated expression of EGLN1 is a self-regulatory response in cocultured MCF-7 cells, in which the cells detect abnormal activity of HIF-1α and compensate by increasing EGLN1 transcription to restore balance. Similar observations of increased mRNA and protein expression of EGLN1 in hypoxic HeLa cells have been reported [51]. The prognostic implications of these hypoxia-induced changes, particularly HIF-1α activity, were demonstrated in a randomized trial involving over 1100 female patients with 15 years of follow-up, revealing that HIF-1α in hypoxic primary breast tumors is strongly correlated with a greater risk of cancer recurrence and death [55].

In addition to the upregulation of the HIF-1 signaling pathway and glycolysis, the downregulation of oxidative phosphorylation in cocultured MCF-7 cells was also noted (Figure 5C). Given the described hypoxia-related shifts, we suspect the occurrence of aerobic glycolysis in ADSC-cocultured MCF-7 samples. This process, also known as the Warburg effect, is associated with the cellular energetic hallmark of cancer, allowing cancer cells to rely on glycolysis for ATP production even when the oxygen supply is adequate [53]. Increased glucose uptake also benefits the proliferation of cancer cells in a multicellular environment, as it limits the glucose supply for tumor-infiltrating lymphocytes that suppress cancer cells [56]. Further consequences include accelerated malignant progression, as well as resistance to drugs and radiation therapy [57].

Another interesting result from GSEA and ORA is the downregulation of the “cell cycle”, “RNA polymerase”, “spliceosome”, and “DNA replication” KEGG pathways, which indicates that the cell cycle and gene expression mechanisms in MCF-7 cells were dysregulated when they were exposed to ADSCs. These changes might indicate cell cycle arrest and inhibited proliferation in cocultured MCF-7 cells. However, our previous findings and other published reports have shown that ADSCs do not affect the proliferation rate of cocultured MCF-7 cells [12,21].

There is evidence that ADSCs influence the behavior of MCF-7 cells through the secretion of cytokines and growth factors while inducing cytokine production in cancer cells [12]. Exposure to high levels of interleukins, such as IL-1β, IL-6, IL-8, IL-15, and TNF-α, leads to many cell responses, including the activation of hypoxia response genes even with a sufficient oxygen supply [58]. Our recent study revealed significant differences in the gene expression levels of IL-6 and AhR between MCF-7 cells cocultured with ADSCs and those monocultured with ADSCs, suggesting that ADSCs may promote the EMT of MCF-7 cells potentially via the AhR/NF-κB pathway [21]. Additionally, exposing cells to TNFα increases HIF-1α activity in normoxic environments via a mechanism in which NF–κB induces the transcription of HIF-1α-stabilizing proteins [59]. Our hallmark enrichment analysis results supported this finding, revealing the upregulation of TNFα signaling via NF–κB (NES = 2.1, FDR < 0.001). Several overexpressed TNF receptors potentially related to this process were also detected in our cocultured samples (data not shown: TNFRSF21: 2^3.5^-fold, TNFRSF19: 2^3.9^-fold).

Upon examining the GSE2034 public gene expression cohort of relapsed and relapse-free breast tumors, several identified hub genes were differentially expressed between the two sample types, as demonstrated by the Wilcoxon rank-sum test. Among them, only ICAM1 was identified by Kaplan–Meier analysis as being associated with reduced cancer recurrence. Interestingly, when we examined the expression of genes included in the Oncotype DX and MammaPrint prognostic panels in our cocultured MCF-7 cells, 19 genes showed statistically significant changes in expression levels. These results underscore the possibility that ADSCs can influence recurrence risk assessments in early-stage breast cancer, thereby affecting therapeutic strategies. Furthermore, overall survival analysis via the TCGA-BRCA dataset revealed that ICAM1 and PGK1 were correlated with mortality, with PGK1 showing effects independent of other clinical factors. ICAM1 expression is exclusive to triple-negative and HER2-positive breast cancer but is induced by proinflammatory cytokines such as TNF–α, IFN–γ, and IL–1β, independent of the tumor hormone receptor status, and is linked to high immune cell infiltration [60]. Its overexpression is also linked to aggressive breast cancer phenotypes with poorly differentiated tumors and positive lymph node involvement and has a significant impact on recurrence-free survival, particularly in patients with ER-negative tumors [61]. PGK1 has been identified as an independent prognostic marker for chemoresistance to paclitaxel treatment in patients with breast cancer, and patients with elevated PGK1 levels who are receiving paclitaxel chemotherapy have significantly shorter survival [62]. Moreover, PGK1 is a transcriptional target of HIF-1α and enhances HIF-1α activity, forming a feedback loop that promotes cancer metastasis [63]. PGK1 inhibition has been shown to reverse the Warburg effect and suppress EMT in both breast cancer and ovarian cancer [63,64].

One of the limitations of this work is that only a single breast cancer cell line (MCF-7) was used with a limited sample size. A more comprehensive investigation utilizing additional breast cancer cell lines as well as various ADSC sources is essential to validate these findings across different breast tumor subtypes. Additional experiments involving the interactions between ADSCs and non-cancerous cells are also crucial to better understanding their effects within a more complex, multi-cellular environment. Moreover, our study only analyzed gene expression profiles at a single time point. This only allowed us to identify transcriptomic patterns that represented ADSCs’ early effects on MCF-7 cells while limiting our ability to fully capture the prognostic relevance between gene expression and relapse or mortality. This limitation is underscored by the observation that while some genes are differentially expressed across cancer stages and relapse statuses, they may not directly contribute to long-term outcomes. Therefore, a more comprehensive time series analysis with extended coculture durations is needed. Furthermore, a larger-scale study incorporating clinical variables alongside gene expression data is essential to account for confounding factors and provide a clearer understanding of the relationship between gene expression and breast cancer relapse for patients receiving ADSC-based therapies. Most importantly, additional studies utilizing molecular techniques, such as RT-qPCR and protein expression assays, are essential, as RNA-seq analysis results can be sequence processing, normalization, and quantification pipelines, as well as the thresholds applied to identify differentially regulated genes and pathways. Confirmation of gene expression changes through these techniques will provide more robust and conclusive insights into their underlying mechanisms.

## 5. Conclusions

In this study, we cocultured MCF-7 breast cancer cells with ADSCs and implemented RNA-seq analysis techniques to investigate the transcriptomic alterations in MCF-7 cells. The results of functional enrichment and coexpression analyses supported the notion that MCF-7 cells cocultured with ADSCs progress toward a more aggressive state, as evidenced not only by the upregulation of ECM remodeling factors but also by the enrichment of the HIF-1 signaling pathway and aerobic glycolysis. The exposure of MCF-7 cells to ADSCs also induced significant changes in the expression levels of genes that are strongly associated with relapse probability and mortality in breast cancer patients. Changes in their expression are also consistent with those observed in the early stages of cancer toward more malignant stages when validated using the GEO and TCGA-BRCA databases. However, it is essential for future research to include both in vitro and in vivo models to confirm these bioinformatics findings and to assess whether these effects take place in clinical or experimental settings.

## Figures and Tables

**Table 1:** Summary of sequence processing results.

Treatment	Sample ^a^	Raw Reads (Millions)	Reads after Filtering (Millions)	Read Mapping (%) ^b^	Gene Assignment (%) ^c^
MCF-7 coculture with ADSCs	CC1	81.07	75.72 (93.4%)	97.01	73.2
	CC2	91.62	85.83 (93.7%)	96.49	72.1
	CC3	83.25	77.43 (93.0%)	96.61	72.5
MCF-7 mono-culture	CT1	90.2	84.26 (93.4%)	96.64	72.9
	CT2	91.56	85.50 (93.3%)	96.51	72.8
	CT3	89.05	83.32 (93.6%)	96.51	71.2

^a^ CC: coculture; CT: control; ^b^ read mapping: % of reads mapped to the reference genome; ^c^ gene assignment: % of reads assigned with gene IDs.

## Data Availability

The raw sequencing data generated in this study were deposited in the NCBI Sequence Read Archive (SRA) under BioProject ID PRJNA1161137 (Biosample SAMN43771358–SAMN43771363).

## Supplementary Materials

The following supporting information can be downloaded at https://www.mdpi.com/article/10.3390/cimb46110702/s1, Figure S1: Quality control results. (A) Averaged Phred quality scores across base positions of reads in all samples. (B) Percentage of unique and duplicated sequencing reads in each sample. A duplication rate ranging from approximately 50 to 60% is expected for RNA-seq. (C) Sequence counts according to the GC content. (D) Coverage profile along genes (CC: cocultured MCF-7, CT: control, i.e., mono-cultured MCF-7, F: forward reads, R: reversed reads); Figure S2: ORA GO term results for the *Coral2* module gene sets, DEGs, and intersection genes: (A) biological process, (B) molecular function, and (C) cellular components; Figure S3: Visualization of the HIF-1 signaling pathway generated using *pathview* (the node colors represent the standardized expression value: red indicates upregulation, blue indicates downregulation, gray indicates no significant changes, and white denotes undetected genes); Figure S4: Expression profile of prognosis genes in the (A) Oncotype DX and (B) MammaPrint gene panels (FDR < 0.001: ***, FDR < 0.01: **, FDR < 0.05: *). Table S1: Characteristics of samples from the GSE2034 dataset used for analysis; Table S2: Characteristics of samples from the TCGA-BRCA dataset used for analysis; Table S3: Wilcoxon rank-sum test results of differential expression of genes between cancer stages and T stages (highlighted in red are genes with *p* < 0.05 in at least one comparison).
